# α7nAChR alleviates cognitive impairment by promoting autophagy in astrocytes via CaMKK2/AMPK/mTOR pathway in sepsis-associated encephalopathy

**DOI:** 10.3389/fimmu.2026.1890443

**Published:** 2026-09-04

**Authors:** Yuesong Liu, Fuhua Wang, Lu Wang, Siqing Wang, Jinyan Xing

**Affiliations:** 1 Department of Critical Care Medicine, The Affiliated Hospital of Qingdao University, Qingdao, China; 2 Qingdao Medical College, Qingdao University, Qingdao, China

**Keywords:** astrocytes, autophagy, neuroinflammation, sepsis-associated encephalopathy, α7 nicotinic acetylcholine receptor

## Abstract

**Background:**

Sepsis-associated encephalopathy (SAE) is a syndrome of cerebral dysfunction secondary to sepsis. Although the α7 nicotinic acetylcholine receptor (α7nAChR) plays a pivotal role in the SAE, the specific mechanisms by which it mediates neuroinflammatory responses and contributes to pathological injury in SAE remain unclear.

**Methods:**

The serum α7nAChR level was measured in all included patients. The ROC curve was used to analyze the α7nAChR level for predicting the risk of SAE. The sepsis rat model was constructed by cecal ligation puncture (CLP). The α7nAChR was activated and inhibited by PNU282987 and Methyllycaconitine (MLA) respectively. Cognitive function and neuronal damage were evaluated using behavioral experiment, Nissl staining, Golgi staining and Electron microscopy. Autophagy was examined by Western blot (WB) and transmission electron microscope (TEM). *In vitro* experiments, lipopolysaccharide (LPS) was used to construct the model of U118. After knockdown and overexpression of α7nAChR, the autophagy proteins level was detected by western blot and the level of cell supernatant inflammatory factors were detected by ELISA. Intracellular calcium concentration was evaluated by flow cytometry.

**Results:**

The serum α7nAChR levels in SAE patients were lower than that in the healthy controls and sepsis patients, and the its AUC for predicting the risk of SAE was 0.79 (95% CI, 0.68–0.90). Activation of the α7nAChR elevated neurobehavioral scores, increased the number of Nissl bodies and synapses, and improved dendritic spine morphology in rats. Activation of α7nAChR weakened the activation of astrocytes, thereby reducing the release of IL-1β, IL-6 and TNF-α. The activation of α7nAChR inhibited the level of P62, up-regulated the expression of LC3. *In vitro*, α7nAChR promoted LPS-induced autophagy in U118 cells, increased intracellular calcium (Ca^2+^) concentrations, elevated p-CAMKK2/CAMKK2 and p-AMPK/AMPK levels, and decreases p-mTOR/mTOR levels.

**Conclusion:**

α7nAChR can alleviate cognitive dysfunction in SAE by activating the Ca^2+^/CaMKK2/AMPK/mTOR signaling pathway and promoting autophagy in astrocytes.

## 1 Introduction

Sepsis-associated encephalopathy (SAE) is a critical public health challenge that poses a significant threat to human health with increased morbidity and mortality (1, 2). Cognitive dysfunction is one of the main clinical manifestations of SAE (3–5). The hippocampus is considered one of the key regions related to the cognitive deficits in the SAE (5). While neuroinflammation (6, 7), autophagy (8, 9), neuronal injury (10, 11) have been implicated in the pathogenesis of SAE, the underlying mechanisms remain incompletely understood due to their multifactorial nature and complex interplay.

As key regulators of the nervous system, astrocytes actively modulate neuroinflammatory responses and synaptic homeostasis, and these functions are frequently compromised in SAE-induced neuronal pathology (7, 12, 13). Astrocyte-derived synapse-modulating factors, including tumor necrosis factor-alpha (TNF-α), interleukin-6 (IL-6), and interleukin-1 beta (IL-1β), play an indispensable role in regulating synaptic plasticity (14, 15). Recent evidence indicates that defective autophagic flux in astrocytes induces reactive gliosis, which involves both the activation of these cells and increased release of inflammatory mediators such as IL-1β and TNF-α. Collectively, these events drive neurotoxic outcomes (16). In contrast, enhanced autophagic flux in astrocytes not only improves neuronal activity but also reduces apoptosis and enhances neural function (17). Therefore, the mechanism associated with astrocyte autophagy and its role in SAE have great clinical significance, which should be explored.

The α7 nicotinic acetylcholine receptor (α7nAChR) is a homopentameric, ligand-gated ion channel with high calcium (Ca^2+^) permeability. It is widely expressed throughout the central nervous system and mediates essential cognitive functions, including memory, learning, and judgment (18, 19). Notably, research suggests that these pro-cognitive effects are predominantly mediated by α7nAChRs expressed on astrocytes. Unlike their neuronal counterparts, astrocytic α7nAChRs sustain N-methyl-D-aspartate receptor (NMDAR) function via the D-serine/NMDAR signaling axis. They also drive both the recruitment of postsynaptic AMPA receptors and the unsilencing of silent synapses. Through these mechanisms, they potentiate astrocyte–neuron network communication, playing a crucial regulatory role in maintaining cognition and sensory processing (20–22). Beyond cognition, α7nAChRs also serve as clinically relevant markers of cholinergic anti-inflammatory pathway (CAP) activity in sepsis, with higher α7 expression correlating with better inflammation control and prognosis (23). Emerging evidence implicates α7nAChR in the pathogenesis of SAE, but its potential mechanism remains unclear (24). Activating α7nAChR has been reported to induce autophagy in macrophages (25, 26), and to exert protective effects across multiple disorders via the AMPK-mTOR-p70S6K pathway (25, 27). However, whether autophagy is involved in α7nAChR-mediated influence on SAE remains unclear.

Here, we investigated the effect of α7nAChR on SAE and its molecular mechanism *in vitro* and *in vivo*.

## 2 Materials and methods

### 2.1 Collection of patient serum samples

Samples from the peripheral blood from 27 SAE patients (conformed to the Sepsis3.0 diagnostic criteria and sepsis-associated encephalopathy diagnostic criteria) and 34 sepsis patients (conformed to the Sepsis3.0 diagnostic criteria but not conformed sepsis associated encephalopathy diagnostic criteria) and 20 age-matched control subjects were analyzed by ELISA. The acute physiology and chronic health evaluation II (APACHE II) score of each group was recorded to reflect the severity of patient’s disease and the sequential organ failure assessment (SOFA) score was used to assess the severity of sepsis. All samples were obtained from June 2023 to December 2023, sepsis patients and sepsis-associated encephalopathy patients admitted to the Intensive Care Department of the Affiliated Hospital of Qingdao University, as well as healthy examination subjects in the Health Examination Center. Diagnosis of sepsis-associated encephalopathy was carried out by an accredited physician. Blood sampling was performed only after written informed consent was obtained from all enrolled patients.

### 2.2 Enzyme linked immunosorbent assay

Blood collection was performed from the healthy controls and patients. The coagulated blood was centrifuged at 3000 rpm for 20 minutes and the supernatant was collected as serum. Cell culture medium was collected and centrifuged to remove precipitates. The concentration of α7nAChR, TNF-*α*, IL-1*β*, IL-6 extracts in serum and cell culture media was measured by using ELISA kits (Elabscience, China).

The rat hippocampal tissues were homogenized in cold PBS and the supernatants were collected and stored at −80 °C until processing. The levels of interleukin (IL)-6 and tumor necrosis factor (TNF)-α in the brain were analyzed using rat ELISA kits (Elabscience, China) according to the manufacturer’s instructions.

### 2.3 Animals

SD rats (male, 6-8weeks, 200 ± 20g) were purchased from Jinan Pengyue Laboratory Animal Company (Jinan, China). The rats were housed in the Medical Animal Laboratory of the Affiliated Hospital of Qingdao University and had free access to water and food. All experimental animals were acclimated for 7 days before conducting experiments. The animal experiment was approved by the Animal Ethics Committee of the Affiliated Hospital of Qingdao University and was conducted according to the National Institutes of Health guidelines (NIH publication no. 85–23, revised 1996). The animal ethics approval number is QYFYWZLL30882.

### 2.4 Cecal ligation and puncture surgery

SAE was induced by cecal ligation and puncture conducted as previously described with a subtle modification (28). Rats were anesthetized by isoflurane inhalation and placed on the operating table (The initial inhalation concentration was 0.5%, and the maintenance concentration was 1.5%.). A wound of about 1-1.5cm was incised in the midline of the abdomen of the rats, then the cecum was isolated. After ligating the cecum with surgical sutures, a single cecum puncture was performed with a 22G sterile needle, followed by gentle pressure on the cecum to leak a small amount of feces. Then the cecum was moved back to the abdominal cavity and the incision was closed. Resuscitation fluid (37 °C, 0.9% NaCl, 50 mL·kg^-1^, i.p.) was given postoperatively. In the sham operation group, only laparotomy was performed without cecal ligation or puncture.

### 2.5 Drug administration

PNU282987(3.0 mg/kg, MedChemExpress, USA), an α7nAChR agonist, was intraperitoneally injected 0.5 h before the CLP procedure. PNU282987 dose was modified based on previous studies (29). The same volume of 0.9% sodium chloride solution was given to the sham and CLP rats. Methyllycaconitine (MLA) (3.0 mg/kg, MedChemExpress, USA), an α7nAChR antagonist, was intraperitoneally injected 0.5 h before the injection of PNU282987 (29).

### 2.6 Neurobehavioral scores

Neurobehavioral scoring was used employed to evaluate symptoms consistent with sepsis-related encephalopathy in rats. The health status of experimental mice was scored by the following five signs: corneal reflex, auricular reflex, rightward turn reflex, tail-flick reflex, and escape reflex. Each reflex was scored as follows:2 points: Presence within 1 second; 1 point: Presence between 1 and 10 seconds; 0 points: Absence. The total score for each animal was calculated by summing the points across all reflexes. The presence of the above reflexes within 1 second was recorded as 2 points; the presence of the reflexes between 1 and 10 seconds was recorded as 1 point, and the absence of the reflexes was recorded as 0 points, and the total score for each group was calculated.

### 2.7 Open field test

Exploratory activity and anxiety-like behavior were measured using a standard open field apparatus (100*100*50 cm, Beijing Zhongshi Dichuang Technology Development Co., Ltd., Beijing, China), as described previously (10). Initially, each rat was placed in the center of the open field. The center zone was a square area with a distance of 10 cm from each wall. The travelling distance, travelling speed, and duration in the center zone were recorded for 5 minutes with a video imaging system.

### 2.8 Y-maze

Working memory and exploratory activity were measured using a Y-maze apparatus (arm length:60 cm, arm width: 10 cm, wall height: 30 cm, Beijing Zhongshi Dichuang Technology Development Co., Ltd., Beijing, China). Each rat was placed in the central area. The number of entries into the arms and alternations were recorded for 10 minutes with the EthoVisionXT video-imaging system. Working memory was calculated as the number of correct alterations/the number of total new arm entries, as described in a previous paper (30).

### 2.9 Nissl staining

Brain samples were perfused with sterile saline, fixed overnight in 4% phosphate-buffered formaldehyde, and then dehydrated using graded sucrose concentrations, 10 - μm - thick sections were prepared for staining (Leica CM 1850, Leica Microsystems AG, Wetzlar, Germany), stained with 0.1% cresyl violet for 10 min and then gradient elution of graded ethanol concentration was used to dehydrate. Finally, the sections were immersed in xylene for 3 min twice and neutral balsam was used to seal them (31).

### 2.10 Golgi staining

Brains were placed directly into Golgi solution (1 g potassium chromate, 1 g mercuric chloride, 0.8 g potassium chloride and 100 ml double-distilled water) where they remained in a bottle kept in the dark for 6 weeks. Thereafter, the solution was exchanged sequentially in 10%, 20% and 30% sucrose solutions in light-protected jars to aid in maintaining histological structure. The brains were sectioned to a thickness of 100 μm with a vibratome and placed onto gelatin-coated glass slides. After rinsing with double-distilled water, slides were incubated in ammonium hydroxide for 30 min. Following a water wash, the slides were incubated for 30 min in a black and white film developer diluted 1:9 with water and then rinsed with final double-distilled water. Slides were mounted with resin and cover-slipped. All subsequent quantitative analyses, which involved repeated measurements, were conducted using ImageJ software in an unbiased manner without bias towards group designation (32).

### 2.11 Transmission electron microscopy

Hippocampal tissues were fixed with 2.5% glutaraldehyde for 24h at 4 °C. After washing with PBS, subsequently, the tissues were fixed with 1% OsO4 for 2 h at room temperature, then dehydrated in ethanol, embedded in Epon-Araldite resin for penetration and placed in a model for polymerization. After the semi-thin section of the samples was used for positioning, an ultrathin section was made and collected for microstructure analysis. The sections were then counterstained with 3% uranyl acetate and 2.7% lead citrate. Sections were observed with an HT7800 transmission electron microscope (Hitachi, Ltd., Tokyo, Japan) (33).

### 2.12 Cell culture, transfection and treatment

The human astrocytoma cell line (U118) was purchased from Procell Co., Ltd. (Procell, China) and cultured in DMEM medium supplemented with 10% fetal bovine serum (Procell, China), 100 μg/ml streptomycin, and 100 μg/ml penicillin according to the instructions provided by Procell. The cells were maintained in an incubator at 37 °C with a 5% CO_2_ atmosphere. The medium was changed every other day, and the cells were passaged using 0.25% trypsin/EDTA (Hyclone). Transfection procedures were carried out according to the manufacturer’s protocol. Briefly, the cells were grown in 6 - well plates for 24 hours and then transfected with the corresponding lentiviruses. The cells were cultured in medium containing 10% FBS for 48 hours, and then the transgene expression was tested or downstream experiments were performed. The lentiviruses were purchased from GeneChem (Shanghai, China). Subsequently, the U118 cells were stimulated with 1 μg/ml of lipopolysaccharide (LPS) for 24 hours.

### 2.13 qRT-PCR

Total RNA from U118 cells was isolated using TRIzol reagent (Invitrogen, Carlsbad, CA, USA) and was reverse transcribed into cDNA using ReverTra^®^ Ace qPCR RT Master Mix with gDNA Remove kit (Vazyme, China). The RT-qPCR was performed using SYBR^®^ Green Real-time PCR Master Mix (Vazyme, China) and data were analyzed using the 2−ΔΔCt method. The primer sequences were as follows: α7nAChR (forward: TGACTGTCTTCATGCTGCTTGTG; reverse: TGCTGGCGAAGTATTGTGCTATC), GAPDH (forward: AAGTTCAACGGCACAGTCAAGG; reverse: GCAATACTCAGCACCAGCATCAC) (34).

### 2.14 Western blot

Brain tissues and cells were homogenized with RIPA lysis buffer including protease inhibitor and centrifuged at 12000 rpm for 15 min at 4 °C. The supernatants were then collected as total protein, and their final concentrations were determined using the bicinchoninic acid protein assay kit. After being heated for 10 min, equal amounts (30 μg) of denatured protein were separated by 12% SDSPAGE and transferred to polyvinylidene difluoride (PVDF) membrane (Millipore, USA). Membranes were blocked with 3% bovine serum albumin for 1 h at room temperature, incubated with primary antibodies overnight at 4 °C, and subsequently washed by TBST three times. Membranes were then incubated with horseradish peroxidase conjugated secondary antibodies (1:2000, Servicebio, China) for 1 h at room temperature and washed in TBST three times before being detected using electrochemiluminescence (ECL) and X-ray films. The densities of blots were examined using ImageJ software (32). Primary antibodies used in the study were as follows: α7nAChR (1:1000, abcam, UK), GFAP (1:1000, Servicebio, China), (LC3B, 1:1000, Zen BioScience Co, China), (P62, 1:1000, Zen BioScience Co, China), p-CaMKK2 (1:1000, CST, USA), CaMKK2 (1:1000, CST, USA), p-AMPK (1:1000, CST, USA), AMPK (1:1000, CST, USA), p-mTOR (1:1000, CST, USA), mTOR (1:1000, CST, USA), GAPDH (1:1000, Servicebio, China), β-actin (1:1000, Servicebio, China).

### 2.15 Immunofluorescence

After being anaesthetized, animals were perfused transcardially with phosphate-buffer saline (PBS) and 4% paraformaldehyde (PFA) successively. Brains were removed and fixed in PFA at 4 °C for more than 24 h. Thereafter, brains were immersed in 30% sucrose for dehydration and then cut into 30-μm-thick slices on a cryostat. Tissue sections were incubated with 0.5℅ Triton X-100 for 20 min for permeation and subsequently immersed in 3℅ bovine serum albumin for 1 h at room temperature for blocking. The sections were then incubated with primary antibodies (1:200) overnight at 4 °C. After being washed with PBS three times, the sections were incubated with secondary antibodies (1:1000) for 1 h at room temperature. Following rinses in PBS, the sections were stained with DAPI. Images were ultimately acquired with the aid of the laser-scanning confocal microscope (Olympus) and analyzed using ImageJ software (32). The percentage of positive area for each channel was quantified using ImageJ (NIH, USA) following threshold-based selection of positive staining areas, with background subtraction and consistent threshold settings applied across all images; at least three non-overlapping fields per section were analyzed blindly.

### 2.16 Measurement of cytosolic Ca^2+^ concentration

Cytosolic Ca^2+^ concentration was measured with Fura RED AM (MCE, China) according to the manufacturer’s instruction. Briefly, cells were washed three times with PBS and incubated in 500 μL of Fura RED AM (5 μM final concentration) in PBS at 37 °C for 30 min, and then washed three times again with PBS and incubated for another 20 min in PBS containing 1% FBS, to ensure that Fura RED AM was completely converted into Fura RED AM. Finally, cells were digested with 0.25% trypsin without EDTA and washed with PBS. The fluorescent intensity was detected using flow cytometry (35).

### 2.17 Statistical analysis

All quantitative analyses were performed with the researcher blinded to the condition. Data were expressed as the mean ± SEM and were analyzed using GraphPad Prism 8 software (GraphPad Software, La Jolla, CA, USA). After the data normality test was performed, the differences between different groups were evaluated by unpaired Student’s t tests and Mann-Whitney U tests or ANOVA for multiple comparisons, one-way ANOVA analysis with Tukey’s *post hoc* test. Spearman’s rank test was used to assess the correlations for univariate analyses. Receiver operating characteristic (ROC) curve was used to assess the performance of each test. A two-tailed *p* < 0.05 was considered statistically significant.

## 3 Results

### 3.1 Serum α7nAChR expression was down-regulated in the patients with sepsis-associated encephalopathy

A total of 81 patients were included in the study. Among them, 27 patients were in SAE group and 34 patients were in sepsis group. There were no significant differences in terms of gender and age. between the two groups had no significant differences. Body temperature, heart rate, and SOFA score also exhibited no significant difference between two groups (*p* > 0.05). Interestingly, the APACHE II score (*p* < 0.01), length of the ventilator (*p* < 0.05), length of ICU stay (*p* < 0.01) and mortality at 28 d (*p* < 0.05), but not 60d mortality in SAE group was significantly higher when compared with the sepsis group. (**Supplementary Table 1**).

**Figure 1:**
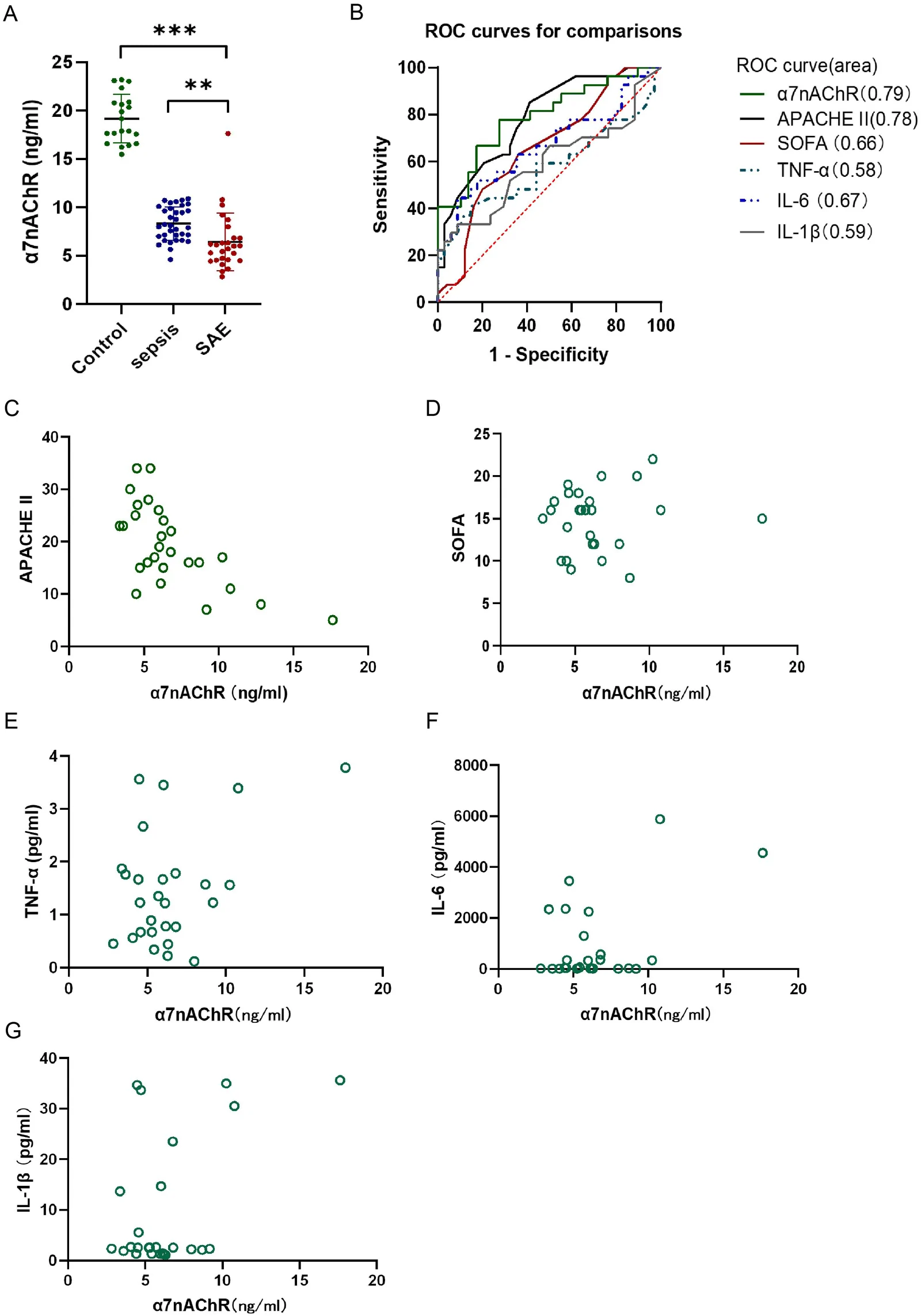
Serum α7nAChR expression was down-regulated in the patients with sepsis-associated encephalopathy. **(A)** The change of α7nAChR levels in peripheral blood of healthy patients, sepsis patients, and SAE patients was detected by ELISA. Healthy controls (n = 20) and sepsis patients without encephalopathy (n = 34), and sepsis related encephalopathy (n = 27). Data are presented as the mean ± SD. **p < 0.01, ***p < 0.001, one-way ANOVA with Tukey’s *post hoc* test. **(B)** The area under ROC curve of SOFA score, APACHE II score, peripheral blood α7nAChR, and TNF-*α*, IL-6, and IL-1*β* of SAE patients. α7nAChR has the largest area under the curve. Pearson correlation analysis between α7nAChR and APACHE II score **(C)**, SOFA score **(D)**, TNF-*α* **(E)**, IL-6 **(F)**, IL-1*β* **(G)** in patients with sepsis associated encephalopathy.

To clarify the change of serum α7nAChR in SAE patients, we compared the serum α7nAChR concentration between the peripheral blood of healthy controls and sepsis patients (19.16 ± 2.52 ng/mL versus 7.29 ± 1.77 ng/mL) (*p* < 0.001). The serum α7nAChR concentration in the SAE was significantly lower compared with the septic group (6.42 ± 2.99 ng/mL versus 7.29 ± 1.77 ng/mL *p* = 0.010) (**Figure 1A**). We further analyzed the predict value of serum α7nAChR, SOFA score, APACHE II score and inflammatory factors such as TNF-*α*, IL-6, and IL-1*β* for the risk of SAE in sepsis patients. (**Figure 1B**). Compared with other parameters, concentration of α7nAChR was the most effective in differentiating SAE from all other conditions ([AUC], 0.79; 95% CI, 0.68–0.90, *p* < 0.001) (**Figure 1B**). Of note, there was a significant negative correlation between α7nAChR and the APACHE II score (r = -0.65, *p* < 0.001) (**Figure 1C**), but no significant correlation was observed between the concentration of α7nAChR and SOFA score, TNF-*α*, IL-6, and IL-1*β* (**Figures 1D–G**).

### 3.2 Activation of α7nAChR alleviated inflammation and improved survival rate

**Figure 2:**
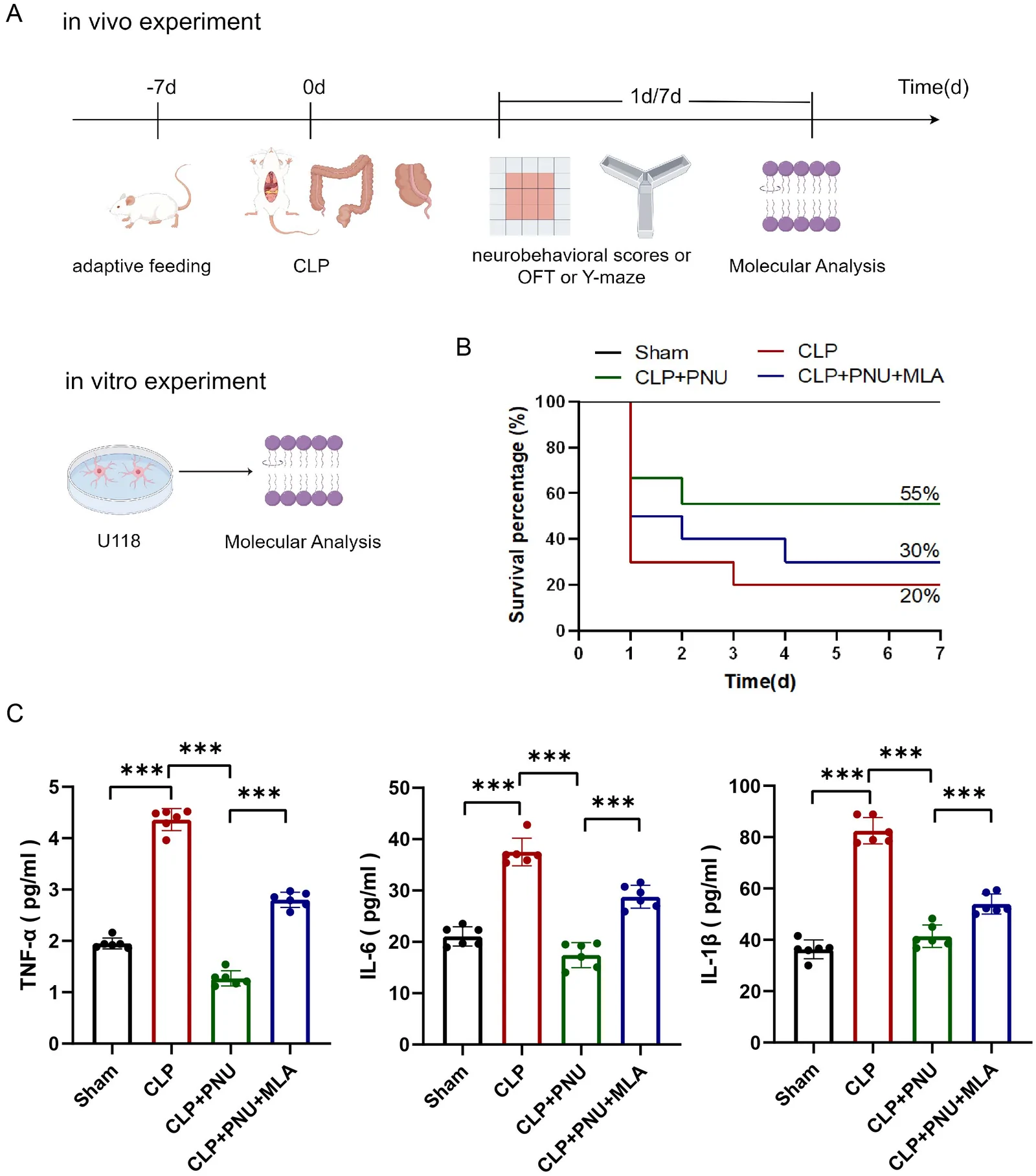
Activation of α7nAChR enhanced survival rate in CLP - induced septic rats and ameliorated inflammation caused by sepsis. **(A)** *In vivo* experiment: The program of septic model preparation and arrangement of neurological score in the present study. *In vitro* experiment: The program of U118 cell assays in the present study is illustrated schematically. **(B)** The survival curve analysis showed the survival rates of rats in each group after modeling. n = 20 rats for each group. **(C)** The expression levels of inflammatory cytokines TNF-*α*, IL-6, IL-1*β* were determined in the hippocampal tissue of rats with sepsis by ELISA. n = 6 for each group. Data are presented as mean ± SD. * *p* < 0.05, **p < 0.01, ***p < 0.001, one-way ANOVA with Tukey’s *post hoc* test.

**Figure 2A** showed the experimental process of the septic rat model. The 7-day survival rates in the sham surgery group, CLP group, CLP+PNU group, and CLP+PNU+MLA group were 100%, 20%, 55%, and 30%, respectively. ELISA results showed that the levels of IL-6 and IL-1β in the hippocampal tissues of CLP rats increased after surgery. The levels of these inflammatory cytokines were significantly decreased in the α7nAChR agonist group and significantly increased in the α7nAChR inhibitor group (*p* < 0.001). The level of TNF-α showed a similar trend (**Figure 2C**).

### 3.3 Activation of α7nAChR ameliorated cognitive dysfunction and alleviated neuronal injury in CLP-induced sepsis rats

**Figure 3:**
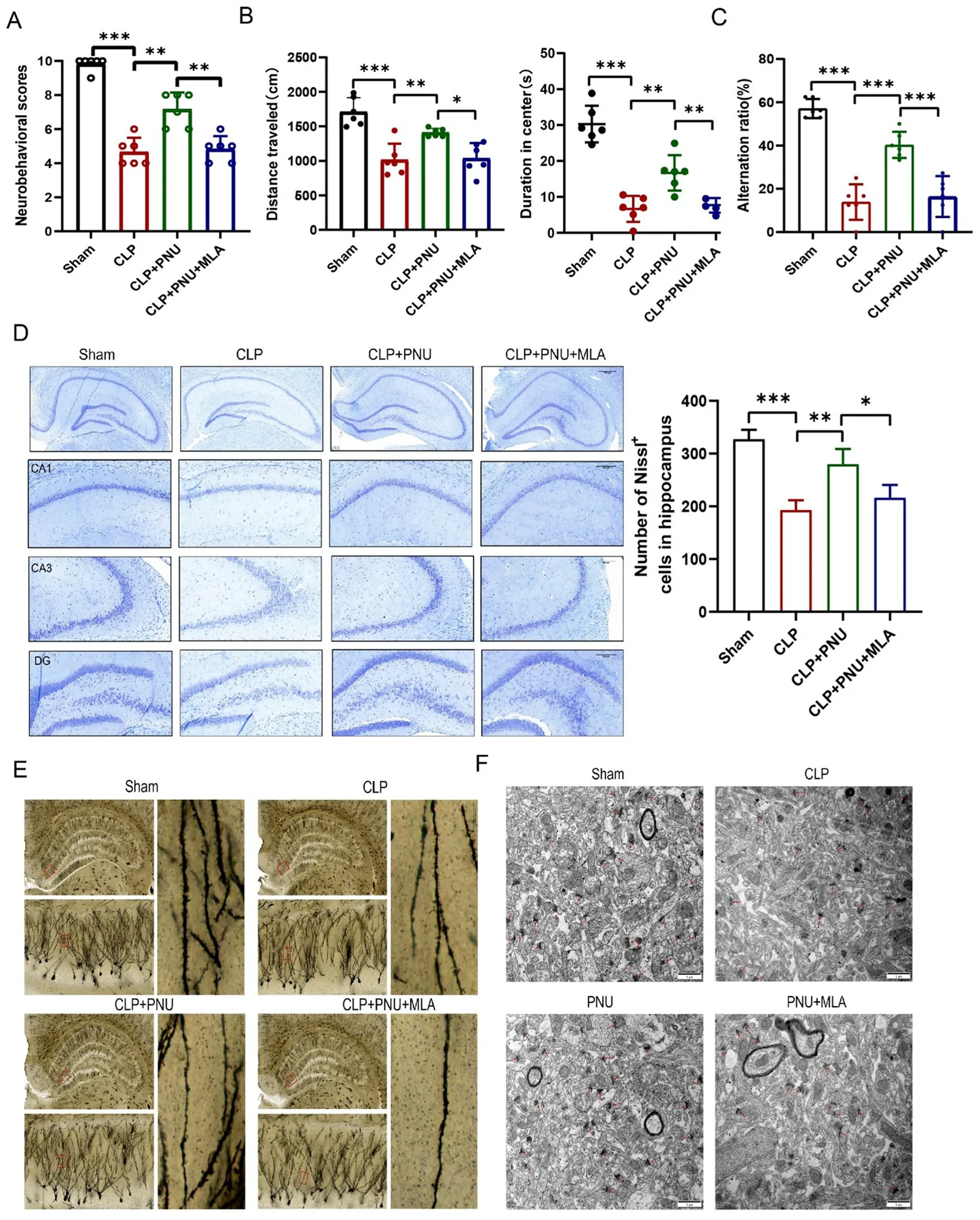
Activation of α7nAChR ameliorated cognitive dysfunction and alleviated neuronal injury of CLP-induced sepsis in rats. **(A)** Neurobehavioral scores reflecting neurological injury in rats. n = 6 rats for each group. **(B)** Rats were subjected to the open field test. Left, total distance travelled, right, time spent in the central area by the rats. n = 5-7. **(C)** The spontaneous alternation rate in the Y-maze. n=6 rats for each group. **(D)** Representative images of Nissl-stained sections of hippocampus from different groups. Scale bar, 500 μm, 200 μm. Right panel, quantification of area and intensity of neuron in the rat hippocampus among different groups. n = 3 rats for each group. **(E)** Representative dendritic spines in hippocampus of four groups (scale bar, 500 μm, 50 μm, 10 μm). **(F)** Representative transmission electron micrographs of synapses in hippocampus (red arrows: synapses; scale bar, 500 nm); **(A–D)** Data are presented as mean ± SD. *p < 0.05, **p < 0.01, ***p < 0.001, one-way ANOVA with Tukey’s *post hoc* test.

To investigate whether α7nAChR has an impact on learning and memory, neurobehavioral scores, open - field test and Y - maze test were conducted after CLP surgery. The neurobehavioral outcome scores of rats in the α7nAChR agonist group were significantly higher than those in CLP group (4.67 ± 0.82 in CLP rats, 7.17 ± 0.98 in PNU282987 rats, and 4.83 ± 0.75 in MLA rats, *p* < 0.01) (**Figure 3A**). The open - field test showed that after the onset of sepsis, the moving distance and the time spent in the central area of rats in the CLP group were significantly lower than those in the sham group. The conditions of rats in the α7nAChR agonist group improved, while those in the α7nAChR inhibitor group were significantly lower than those in the α7nAChR agonist group (**Figure 3B**). The Y - maze test showed that the correct spontaneous alternation rate of the PNU group was significantly higher than that of the CLP group. Compared with rats in the PNU group, rats in the MLA group had significant memory function deficits (**Figure 3C**). These results indicate that activation of α7nAChR significantly improves CLP - induced hippocampus - dependent cognitive dysfunction (learning and memory functions) in septic rats.

Sepsis rats exhibited a reduction in hippocampal dendritic spines, leading to cognitive dysfunction. In Nissl staining, we found that the neuron bodies of in the CLP model were much larger and lighter in color. Specifically, the number of neurons was significantly greater in the PNU group than in the CLP rats. However, MLA mitigated the effect of the α7nAChR agonist (**Figure 3D**). Structural synaptic plasticity was verified by measuring changes in postsynaptic dendritic complexity. Golgi staining was used to observe dendritic spine density and hippocampal morphology (**Figure 3E**). The results showed that compared with CLP rats, the neurite branching, spine generation, and maturation in rats of the α7nAChR agonist group were significantly increased, while these data decreased in the α7nAChR inhibitor group. The results revealed that α7nAChR activation had an improving effect on the quantity and morphological changes of synaptic damage in septic rats. There was a significant increase in synapses in the α7nAChR agonist group (**Figure 3F**). In contrast, CLP rats showed a lower synaptic density (**Figure 3F**). These results indicate that α7nAChR activation improved cognitive dysfunction and synaptic plasticity in CLP-induced sepsis in rats.

### 3.4 Activation of α7nAChR diminished astrocyte activation and restored autophagy in septic rats’ brain

**Figure 4:**
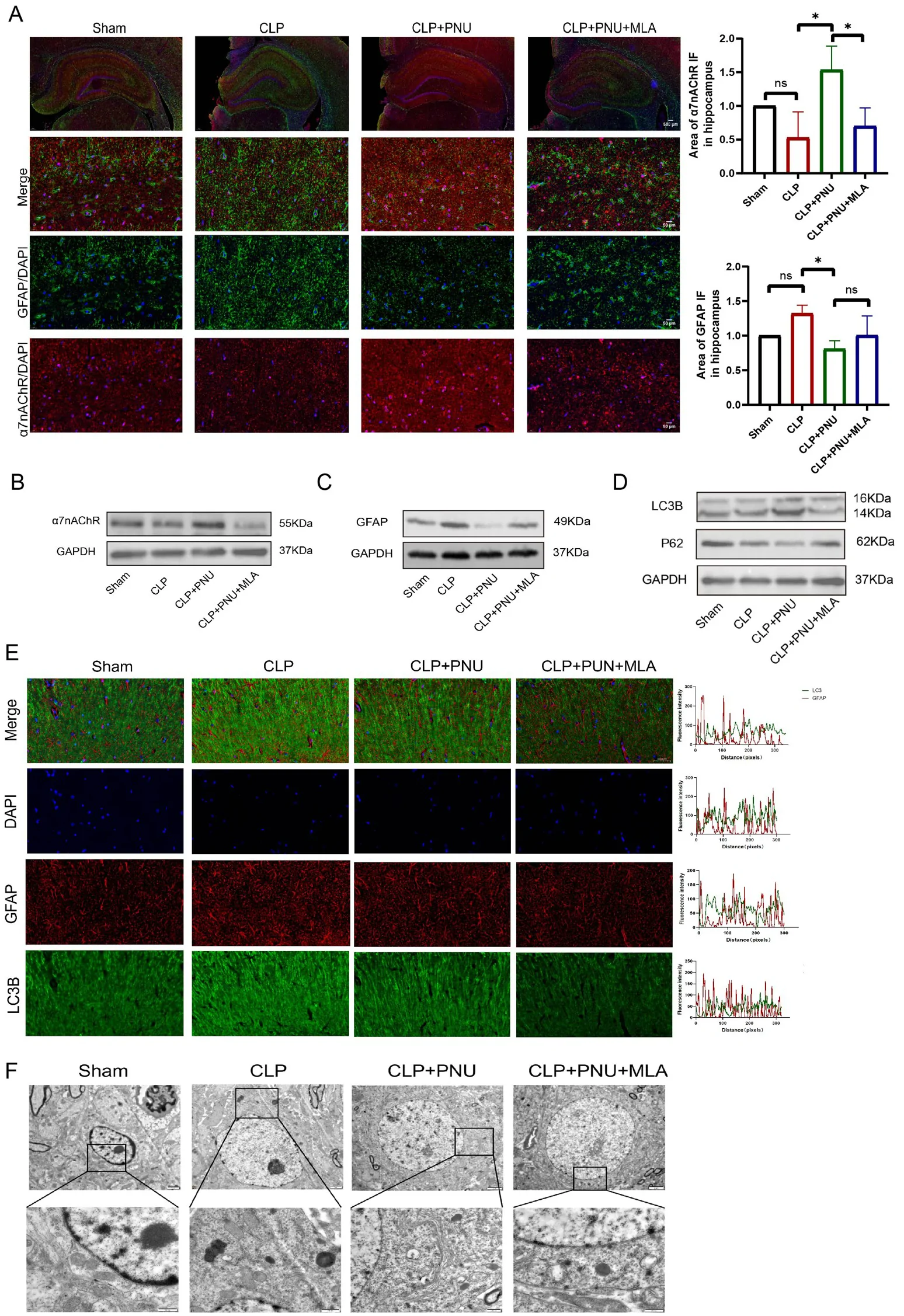
Activation of α7nAChR diminished astrocyte activation and restored autophagy in brains of septic rats. **(A)** Immunofluorescence of GFAP+(green) astrocytes and α7nAChR (red) in hippocampal sections of rat brains (left), white arrows indicate astrocytes, scale bars for different magnifications are as follows: 500 μm, 50 μm. Right panel, quantification of area and intensity of GFAP and α7nAChR in the hippocampi of rats among different groups. n = 3 rats for each group. **(B)** Representative Western blot bands of the α7nAChR expression levels in the rats’ hippocampus among different groups. n = 3 rats for each group. **(C)** Representative western blot bands of the GFAP expression levels in hippocampus of rats. n = 3 rats for each group. **(D)** Representative western blot bands of the LC3B-II, LC3B-I, and P62 expression levels in hippocampus of rats among different groups. n = 3 rats for each group. **(E)** Brain slices of rats in each group rats were immunostained with LC3B (green) and GFAP (red) (complete co-localization) in the hippocampal CA1 region (left), scale bar, 40 μm. **(F)** Representative transmission electron micrographs of the hippocampus (Scale bar, 200 μm) of full image (Scale bar, 1 μm). Data are presented as mean ± SD. *p < 0.05, **p < 0.01, ***p < 0.001, one-way ANOVA with Tukey’s *post hoc* test.

Astrocyte activation is one of the main neuropathological features of SAE. Astrocytes secrete pro - inflammatory factors, which can induce and regulate the degree and outcome of the inflammatory response, thereby controlling neuroinflammation in sepsis (36, 37). We investigated the effect of α7nAChR on the astrocyte response induced by CLP, especially observing its activation. Therefore, western blot and immunofluorescence staining were performed. GFAP and α7nAChR were used to detect astrocyte immunoreactivity in rat hippocampal tissues via immunohistochemistry. In septic rats, GFAP - positive cells showed enlarged cell bodies with thick, retracted processes, which was consistent with the morphology of activated astrocytes as indicated by the white arrows in **Figure 4A**. In contrast, GFAP - positive cells in the sham - operated group showed thin cell bodies with thin and long processes, which was again consistent with the morphological branching of resting astrocytes. The number of GFAP - positive cells in the hippocampus of CLP rats was significantly increased compared with that of sham - operated rats (**Figure 4A**). In the α7nAChR agonist group, the area of GFAP - positive cells in the hippocampus was significantly reduced (**Figure 4A**). Similarly, GFAP protein expression increased in septic rats and decreased in rats treated with the α7nAChR agonist (**Figure 4C**, **Supplementary Figure 1B**, **Supporting Information**). We found that the hippocampal α7nAChR protein level in the CLP group was significantly lower than that in the sham operation group, while it was significantly higher in the PNU group compared with the CLP group (**Figure 4B**, **Supplementary Figure 1A**, **Supporting Information**).

According to the relevant literature the pathogenesis of SAE often involves autophagy. In our present study, the level of LC3B - II in the hippocampal region of CLP rats was higher than that of sham - operated rats; however. In contrast, the level of LC3B - II in rats in the α7nAChR agonist group was further increased compared with CLP rats (**Figure 4D**, **Supplementary Figure 1C**, **Supporting Information**). Compared with the sham group, the content of P62 in the hippocampus of CLP rats reduced significantly (**Figure 4D**, **Supplementary Figure 1D**, **Supporting Information**). α7nAChR is expressed in various types of cells in the CNS. Therefore, to further evaluate whether astrocyte activation is caused by the inhibition of astrocyte autophagy, we performed double immunofluorescence staining of LC3B and GFAP in rat brain slices. Rats in the α7nAChR agonist group showed an increased level of co - localization between LC3B and GFAP, and the intensity of GFAP decreased in rats that underwent CLP (**Figure 4E**). Transmission electron microscopy analysis revealed that the number of autophagosomes in the hippocampus of CLP rats was higher than that in sham - operated rats, and it was further increased in rats in the α7nAChR agonist group after CLP (**Figure 4F**).

### 3.5 Overexpression of α7nAChR promoted autophagy in U118 cells

**Figure 5:**
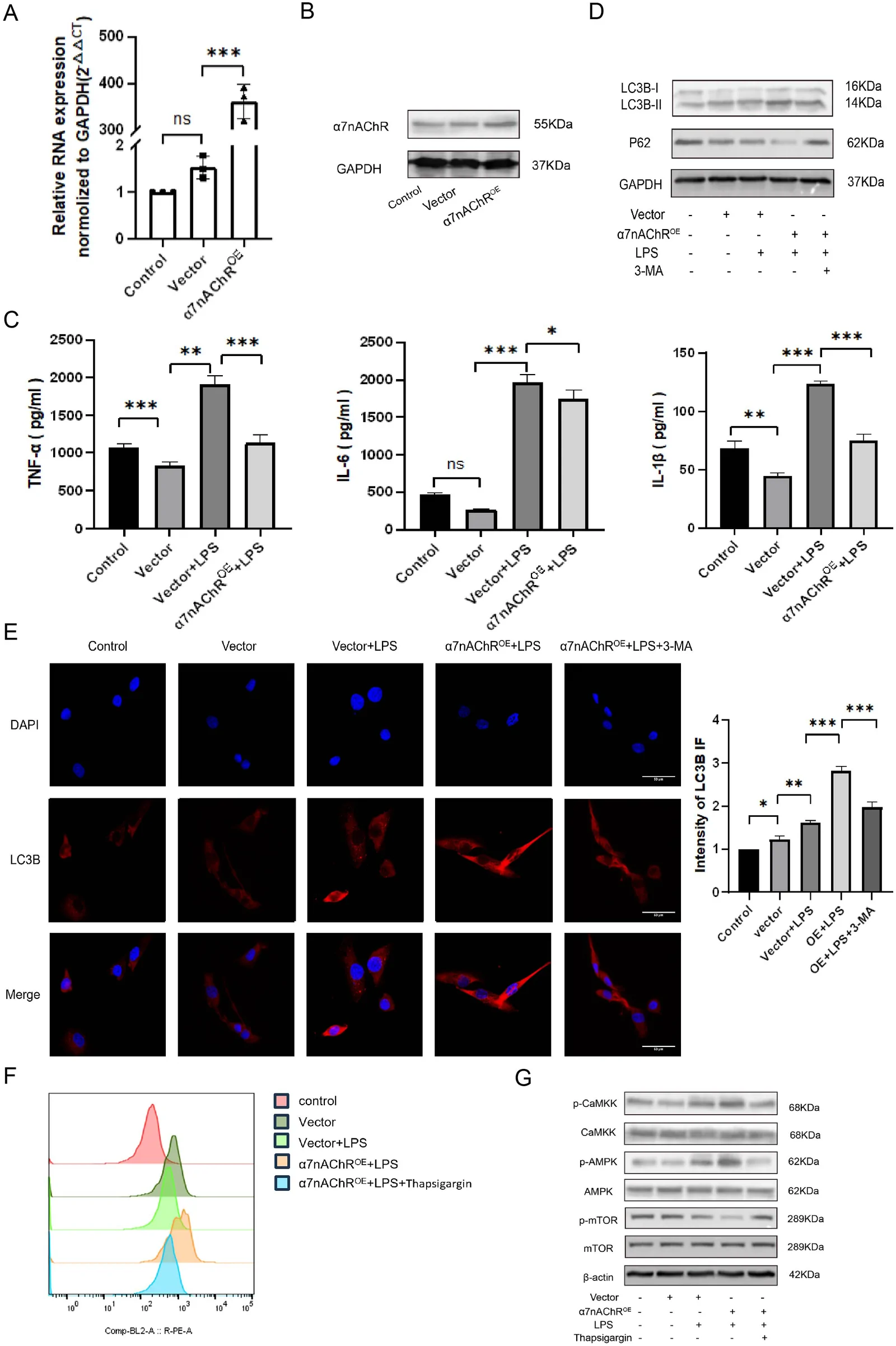
α7nAChR overexpression attenuated inflammation and promoted cell autophagy via Ca^2+^/CAMKK2/AMPK/mTORC1 pathway. **(A)** Relative quantification of α7nAChR expression levels detected by qRT-PCR after transfection with α7nAChR overexpression lentivirus. **(B)** Western blotting of α7nAChR expression levels after transfection with the α7nAChR overexpression lentivirus. **(C)** The expression levels of inflammatory cytokines TNF-*α*, IL-6, IL-1*β* were determined in astrocyte culture media by ELISA in α7nAChR overexpression astrocytes treated with LPS. n = 3 for each group. **(D)** The protein levels of P62, LC3 were determined by Western blot in α7nAChR-overexpression astrocytes treated with LPS (1ug/ml 24h) or 3-MA (2μM 2h), n = 3 for each group. **(E)** U118 cells were immunofluorescently stained with LC3B (red), DAPI (blue), and the astrocytes were simultaneously treated with LPS or 3 - MA, scale bar, 50 μm; right panel, quantitative analysis of intensity of LC3B puncta immunofluorescence **(F)** Intracellular Ca^2+^ levels in U118 cells were analyzed by flow cytometry after staining with the fluorescent probe Fura RED AM. **(G)** Western blots of indicated proteins in cells treated with LPS or Thapsigargin(1μM 6h). Data are presented as mean ± SD. *p < 0.05, **p < 0.01, ***p < 0.001, ****p < 0.0001, one-way ANOVA with Tukey’s *post hoc* test.

To elucidate the regulatory effect of α7nAChR on autophagy in U118 astrocytes, we constructed a lentivirus for α7nAChR overexpression *in vitro*, transfected it into U118, and confirmed the increased level of α7nAChR at the mRNA and protein levels respectively (**Figures 5A, B**, **Supplementary Figure 2A**, **Supporting Information**). Initially, we detected inflammatory factors and found that the levels of inflammatory factors (IL - 6, IL - 1β, TNF - α) decreased after α7nAChR overexpression (**Figure 5C**). When U118 cells with overexpression of α7nAChR were stimulated with LPS, it was found that the expression level of P62 was decreased, and the expression of LC3 was increased (**Figure 5D**, **Supplementary Figures 2B, C**, **Supporting Information**). As shown in **Figure 5E**, the immunofluorescence results also demonstrated that the overexpression of α7nAChR significantly enhanced LPS - mediated LC3B staining. It is therefore necessary to further clarify the underlying mechanism of α7nAChR in activating autophagy. We therefore investigated the status of Ca^2+^/CAMKK2/AMPK/mTORC1 pathway. Both intracellular calcium concentration and CAMKK2/AMPK/mTORC1 pathway-related proteins were detected by flow cytometry and western blot, respectively. After overexpressing α7nAChR in U118 cells, calcium ion concentration and the expression of p-CAMKK2(S511), p-AMPK(T172) and LC3II were increased, while the level of p-mTOR (S2448) and P62 was significantly decreased (**Figures 5F, G**, **Supplementary Figures 2D–F**, **Supporting Information**). These results review that α7nAChR activated cell autophagy via Ca^2+^/CAMKK2/AMPK/mTORC1 pathway.

### 3.6 Knockdown of α7nAChR inhibited autophagy in U118 via Ca^2+^/CAMKK2/AMPK/mTORC1 pathway

**Figure 6:**
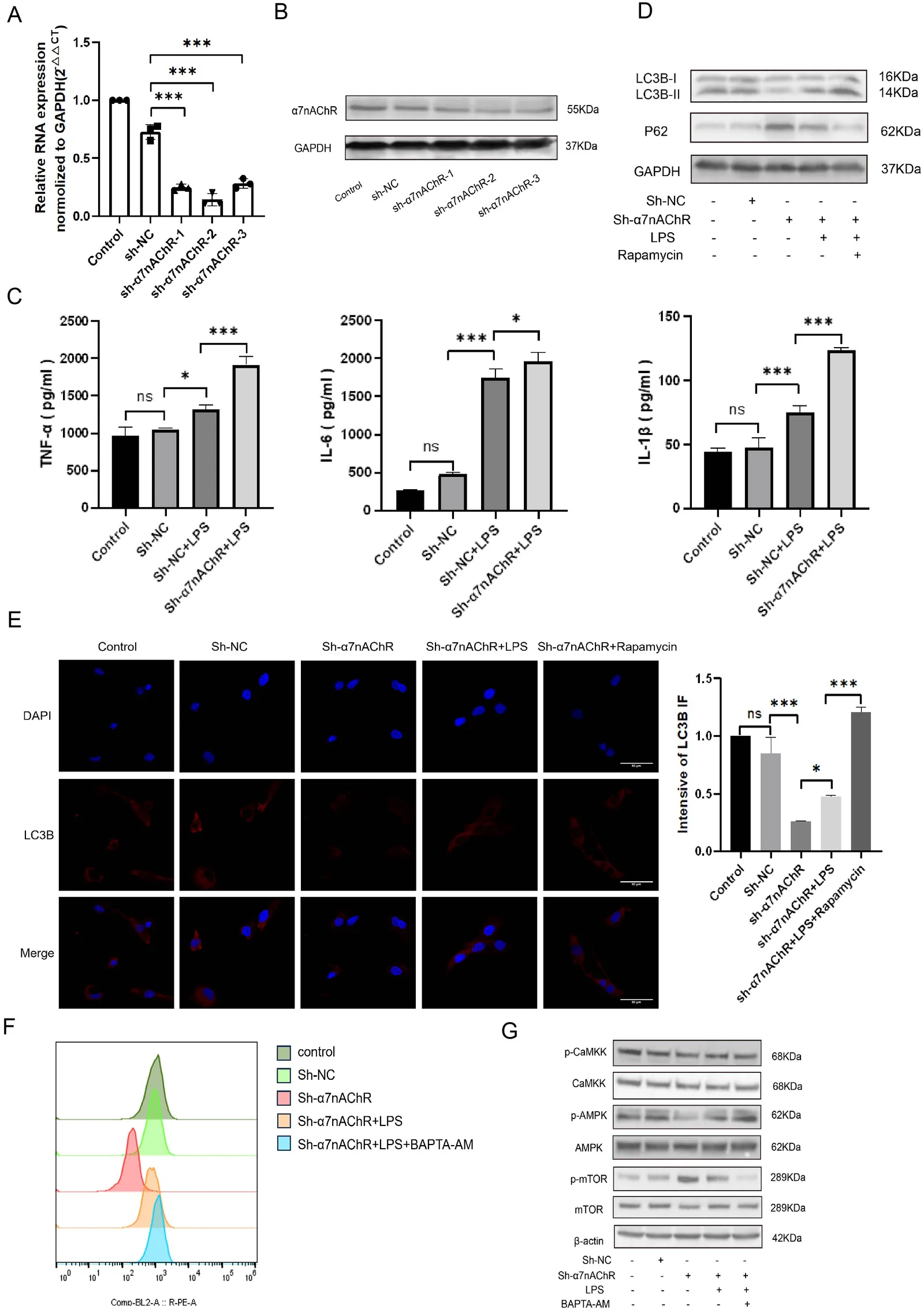
Knockdown of α7nAChR attenuated inflammation and inhibited cell autophagy via Ca^2+^/CAMKK2/AMPK/mTOR1 pathway. **(A)** Relative quantification of α7nAChR knockdown levels was detected by qRT-PCR after transfection with the α7nAChR knockdown lentivirus. **(B)** Western blotting of α7nAChR knockdown levels after transfection with α7nAChR knockdown lentivirus. **(C)** In α7nAChR knockdown astrocytes treated with LPS, the expression levels of inflammatory cytokines TNF - *α*, IL - 6, and IL - 1*β* in astrocyte culture media were determined by ELISA. n = 3 for each group. **(D)** The protein levels of P62, LC3 were determined by Western blot in α7nAChR knockdown astrocytes treated with LPS (1μg/ml 24h) or Rapamycin (10 nM 12h), n = 3 for each group. **(E)** U118 cells were immunofluorescently stained with LC3B (red), DAPI (blue), simultaneously. The astrocytes treated with LPS or Rapamycin, scale bar, 50 μm; right panel, quantitative analysis of intensity of LC3B puncta immunofluorescence **(F)** Intracellular Ca^2+^ levels were analyzed by flow cytometry after staining with the fluorescent probe Fura RED AM in U118 cells. **(G)** Western blotting was performed to analyze the indicated proteins in cells treated with LPS or BAPTA-AM (10 μM 12h). Data are presented as mean ± SD. *p < 0.05, **p < 0.01, ***p < 0.001, one-way ANOVA with Tukey’s *post hoc* test.

To further clarify the role of α7nAChR in autophagy, we constructed an α7nAChR knockdown lentivirus *in vitro*, transfected it into U118 cells, achieving successfully knockdown of α7nAChR expression (**Figures 6A, B**, **Supplementary Figure 3A**, **Supporting Information**). Sh-α7nAChR-2 was selected for the subsequent experiments. ELISA results revealed that after α7nAChR knockdown, the levels of inflammatory cytokines, such as IL-6, IL-1β, and TNF-α, were elevated (**Figure 6C**). Additionally, the results from immunofluorescence and Western blot were consistent. α7nAChR knockdown led to downregulation of LC3 expression (**Figures 6D, E**) and upregulation of P62 (**Figure 6D**, **Supplementary Figures 3B, C**, **Supporting Information**). The flow cytometry results indicated a significant decrease in intracellular calcium ions upon α7nAChR knockdown (**Figure 6F**). Furthermore, our data showed that knockdown of α7nAChR reduces the phosphorylation levels of p-CaMKK2 (Ser511) and p-AMPK (T172), while the level of phosphorylated mTOR (Ser 2448) increases, which is contrary to the results after overexpression of the *α7nAChR* gene (**Figure 6G**, **Supplementary Figures 3D–F**, **Supporting Information**). In summary, knockdown of α7nAChR inhibits autophagy in cells through the Ca^2+^/CAMKK2/AMPK/mTORC1 pathway.

## 4 Discussion

**Figure 7:**
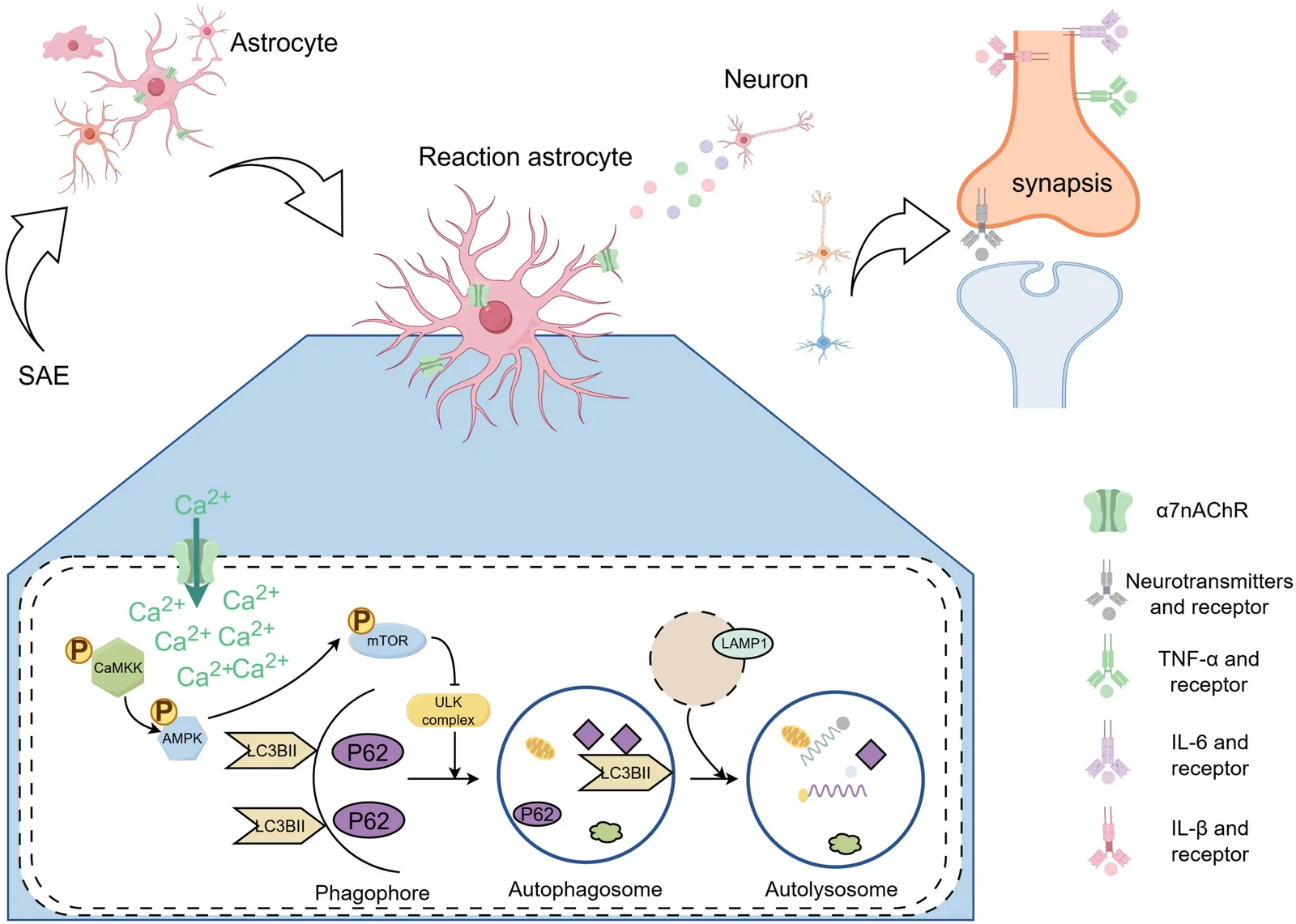
The α7nAChR mitigates neuronal damage during SAE by activating the Ca^2+^/AMPK/CaMKK/mTOR pathway. Researchers propose a mechanistic pathway diagram for α7 intervention in SAE progression. The α7 receptor can elevate intracellular Ca^2+^ levels, which promotes the activation of CaMKK and AMPK to enhance autophagy and alleviate neuronal injury; it can also reduce astrocytic inflammatory responses, thereby mitigating neuronal damage.

Although neuroinflammation, autophagy, and neuronal damage have been reported as the etiologies of SAE, the underlying pathological mechanisms of SAE are very complex, and the exact cause remains unclear. α7nAChR, a pentameric ligand - gated ion channel with high permeability to Ca^2+^, is highly expressed in hippocampal and cortical neurons. Previous studies have shown that α7nAChR plays an important role in various neurodegenerative diseases (38–41). As a key member of the nicotinic receptor family, α7nAChR mediates anti-inflammatory responses in sepsis via the cholinergic anti-inflammatory pathway. In SAE, CAP dysregulation drives neuroinflammation and immune dysfunction (6), while astrocytic α7nAChR activation exerts protective effects through NF-κB inhibition and Nrf2 activation (42, 43). However, little is known about the biological function of α7nAChR in the development and progression of SAE. Our current study showed that α7nAChR promotes autophagy of astrocytes *in vitro* and *in vivo* by regulating the Ca^2+^/CAMKK2/AMPK/mTORC1 pathway (**Figure 7**).

The clinical observation that serum α7nAChR levels were significantly reduced in SAE patients, with an AUC of 0.79, raises two important questions regarding its translational potential. First, whether circulating α7nAChR levels reflect central nervous system expression remains to be fully elucidated. Importantly, hippocampal α7nAChR protein expression has been shown to be significantly decreased in SAE male mice, correlating with cognitive dysfunction (44). Consistent with the observed reduction of hippocampal α7nAChR in SAE rodents, our clinical data showing decreased serum α7nAChR in SAE patients suggest that circulating levels of this receptor may partially mirror central cholinergic deficits. Second, serum α7nAChR shows promise as a non-invasive biomarker for SAE. Notably, in septic patients, peripheral blood mononuclear cell α7 gene expression levels have been shown to correlate directly with vagal heart input and inversely with inflammatory state, disease severity, and clinical outcome, suggesting that α7nAChR expression is a clinically relevant marker for cholinergic anti-inflammatory pathway activity in sepsis (23). Future prospective studies with larger cohorts and longitudinal sampling are needed to validate whether serum α7nAChR levels can predict SAE onset, track disease progression, or guide therapeutic stratification.

We found that rats in the α7nAChR agonist group showed significantly improved clinically relevant indicators, including survival rate and neurobehavioral scores. It was also found that α7nAChR could improve cognitive impairment and synaptic density in CLP rats. There is increasing evidence that sepsis activates astrocytes, leading to the production of a large number of inflammatory cytokines. In addition, astrocyte-mediated neuroinflammation after the onset of sepsis plays a key role in the development of long-term cognitive dysfunction. This experiment confirmed that CLP could induce astrocyte activation and produce pro-inflammatory cytokines, and that astrocyte-mediated neuroinflammation plays a crucial role in SAE.

Autophagy is a highly conserved intracellular degradation pathway involved in the pathogenesis of sepsis-associated encephalopathy (SAE), yet its precise role remains incompletely understood. The process involves conjugation of cytoplasmic LC3-I to phosphatidylethanolamine, followed by conversion to membrane-bound LC3-II, which is subsequently degraded via the p62/SQSTM1 pathway (45). Emerging evidence indicates that autophagy can exert either protective or detrimental effects depending on disease stage, severity, and cell type involved.

In the acute phase, pharmacological inhibition of autophagy using 3-methyladenine (3-MA) attenuates neuroinflammation and improves cognitive function in septic mice (46). PDTC similarly ameliorates SAE by suppressing autophagy and inflammatory responses (47). Conversely, during the recovery phase, appropriate autophagic activity facilitates clearance of damaged organelles. Studies have shown that autophagy in septic brains is significantly inhibited after a brief initial increase, and this sustained inhibition leads to accumulation of damaged organelles, activation of inflammatory complexes, and exacerbation of cognitive dysfunction (48–50).

Microglial autophagy is a key regulator of microglial activation and neuroinflammation in SAE (51), while CXCR5 down-regulation alleviates cognitive dysfunction via modulation of microglial autophagy and p38MAPK/NF-κB/STAT3 signaling (52). In astrocytes, autophagy appears more context-dependent: inhibiting autophagy leads to astrocyte activation and increased release of inflammatory factors (16), whereas upregulating autophagy in astrocytes increases neuronal activity and reduces apoptosis (17). AQP4 aggravates cognitive impairment by inhibiting Nav1.6-mediated astrocytic autophagy (13), while LC-HP-NA activation promotes astrocytic AQP4-related autophagy via α2A-AR and cAMP/PKA signaling, conferring neuroprotection (53). In neurons, SESN2 promotes ULK1-dependent autophagy via AMPK/mTOR (54), and chaperone-mediated autophagy alleviates cognitive impairment by reducing neuronal death (55), yet nuclear autophagy overactivation may also contribute to pathology (56).

Our study demonstrates that sepsis reduces autophagosome formation and LC3B-II levels in rat brain while increasing free p62 levels, confirming autophagy dysregulation. Notably, α7nAChR is widely expressed in astrocytes, the most abundant cell type in the hippocampus. We found that activating α7nAChR promotes astrocytic autophagy, as evidenced by increased LC3B-GFAP co-localization, and concurrently reduces astrocyte activation. Given that astrocytic autophagy upregulation confers neuroprotection while its inhibition exacerbates damage, our findings suggest that α7nAChR-mediated astrocytic autophagy represents a cell type-targeted approach that may tip the balance toward neuroprotection.

Intracellular free Ca^2+^, a second messenger within cells, is regarded as an important regulator of autophagy, and an increase in free cytoplasmic calcium is an effective inducer of autophagy (57). Vitamin D3 compounds, ionomycin, ATP, and thapsigargin inhibit the activity of the mammalian target of rapamycin (mTOR), a negative regulator of autophagy, and induce the massive accumulation of autophagosomes in a Beclin 1- and Atg 7-dependent manner. This process is mediated by calcium/calmodulin-dependent kinase-β and AMP-activated protein kinase (Ca^2+^/CaMKK2/AMPK/mTOR pathway) and is inhibited by ectopic Bcl-2 and the Ca^2+^ chelator BAPTA (58, 59). Calmodulin dependent kinase type β (CAMKKβ, also known as CAMKK2) is involved in the regulation of AMPK and the induction of autophagy in response to changes in intracellular Ca^2+^ levels (60, 61). Membrane fusion events during autophagy require Ca^2+^ (62). The increased expression of α7nAChR in U118 cells (as shown by our data) can elevate the concentration of intracellular free Ca^2+^, thereby activating the Ca^2+^/CaMKKβ/AMPK/mTORC1 pathway and ultimately downregulating the protein levels of LC3B-II. The experimental results of treating astrocytes with a Ca^2+^ mobilizer (Thapsigargin) and a Ca^2+^ chelator (BAPTA-AM) further confirm that Ca^2+^ and the CaMKK2/AMPK/mTORC1 pathway mediate α7nAChR-induced autophagy in astrocytes. The detailed mechanism by which α7nAChR induces an increase in cytoplasmic Ca ^2+^ concentration still needs further investigation.

Several limitations of the present study should be acknowledged. First, the clinical cohort used for validation was relatively small, which may limit the statistical power and generalizability of our findings. Large-scale, multicenter prospective studies are needed to confirm the clinical relevance of our observations. Second, the absence of longitudinal patient follow-up precludes assessment of the long-term prognostic value of the identified autophagy-related markers. Future studies incorporating longitudinal cognitive and functional outcome assessments would be valuable to determine whether these biomarkers predict recovery or chronic cognitive impairment in SAE survivors. Third, while we demonstrated the involvement of the CaMKK2/AMPK pathway in regulating autophagy *in vitro*, we did not perform direct pharmacological inhibition of this pathway *in vivo*. Although Kahweol has been shown to exert protective effects in sepsis-induced acute lung injury via a CaMKKII/AMPK-dependent pathway (63), whether this pathway similarly modulates autophagy and neuroinflammation in the brain during SAE requires further investigation using specific inhibitors such as STO-609 in appropriate animal models. Fourth, regarding our cell model, we acknowledge that U118 MG cells originate from human astrocytoma and carry genetic alterations including PTEN loss (64) and p53 mutation that may influence signaling pathways. While U118 MG cells express astrocytic markers such as GFAP (65) and exhibit similar responses to primary astrocytes in certain contexts (66, 67), findings from this cell line warrant cautious interpretation. Nevertheless, several studies have successfully employed U118 MG cells in parallel with primary astrocytes to corroborate key observations (68). Future investigations should prioritize confirmation of our findings in primary astrocyte cultures and appropriate *in vivo* models of SAE. Despite these limitations, our study provides novel insights into the role of astrocytic autophagy and α7nAChR signaling in SAE pathogenesis and identifies potential therapeutic targets for further investigation.

In summary, our study found that α7nAChR alleviates sepsis-induced neuronal damage and cognitive dysfunction by promoting CaMKK2/AMPK/mTOR-dependent autophagy and inhibiting the inflammatory response of astrocytes. The mechanism may be related to the increase of Ca^2+^ influx in astrocytes mediated by α7nAChR, which provides new insights into the pathological mechanism of sepsis-induced neuronal damage and also offers potential new drug targets for the treatment of SAE.

## Data Availability

The original contributions presented in the study are included in the article/**Supplementary Material**. Further inquiries can be directed to the corresponding author.
