# Supplementary material for: α7nAChR alleviates cognitive impairment by promoting autophagy in astrocytes via CaMKK2/AMPK/mTOR pathway in sepsis-associated encephalopathy
Source: Front Immunol. 2026 Sep 4;17:1890443. doi: 10.3389/fimmu.2026.1890443 (PMC13585608; doi:10.3389/fimmu.2026.1890443)
Supplement: Supplementary file 1 [file DataSheet1.pdf]

frontiers

The International Society for the Advancement of Cytometry (ISAC) has highlighted the importance of including comprehensive methodological information to ensure data reproducibility and reliability. In line with this, Frontiers in Immunology now requires authors to submit a checklist for manuscripts that involve flow or mass cytometry. This checklist helps standardize the reporting process, improving the quality and transparency of published data. By doing so, we support scientific progress, making it easier for other researchers to replicate and validate experiments.

This form should be submitted with any manuscripts using flow or mass cytometry.

Sample/specimen/material description

- [ ] Total blood
- [ ] PBMCs
- [ ] Organ digests

Other The human astrocytoma cell line (U118)

Did the samples suffer any treatment before or after incubation with the antibodies?

- [x] Drug LPS (1ug/ml 24h) or Thapsigargin(1μM 6h) or BAPTA-AM (10 μM 12h)
- [ ] Cell permeabilization ______________________
- [ ] Dye ______________________________________
- [ ] Propidium iodine
- [ ] Not applicable

Other Lentivirus-mediated α7nAChR overexpression and knockdown were established in the U118 cell line

Instrument and antibodies

Name of the Cytometer Attune NxT Acoustic Focusing Cytometer

Antibodies and targets	Fluorochrome/ Metal	Catalog number/Company
the cytosolic Ca(2+)concentrations	Fura Red AM	Cat.HY-D1903/MedChemExpress LLC
e.g. anti-CD4	FITC	Cat. XXX/ XXX ltd.

Data analyses

1. Name of the software FlowJo_V10
2. Reference gating strategy in the manuscript or supplementary material

Gating strategy in (eg Figure X) Figure 5F and Figure 6F
