# Supplementary material for: α7nAChR alleviates cognitive impairment by promoting autophagy in astrocytes via CaMKK2/AMPK/mTOR pathway in sepsis-associated encephalopathy
Source: Front Immunol. 2026 Sep 4;17:1890443. doi: 10.3389/fimmu.2026.1890443 (PMC13585608; doi:10.3389/fimmu.2026.1890443)
Supplement: Supplementary file 2 [file Table1.docx]

Table S1 Participant Characteristics

| Characteristics | Sepsis group (n = 34) | SAE group (n = 27) | P |
| --- | --- | --- | --- |
| Sex, (male/female) | 20/14 | 17/10 | 0.747 |
| Age, median (IQR), y | 70 (21-90) | 76（40-90） | 0.144 |
| Temperature,℃ | 36.57±0.33 | 36.84±0.70 | 0.058 |
| Heart rate/min | 85.59±14.19 | 90.11±18.78 | 0.288 |
| Shock (yes/no) | 18/16 | 11/16 | 0.287 |
| APACHE II score^a^ | 16.74±6.24 | 21.42±6.46 | *0.006* |
| SOFA score^b^ | 7.30±4.39 | 8.36±4.23 | 0.343 |
| Length of ventilator，d | 5.68±4.60 | 76.2±182.5 | 0.049 |
| Length of ICU stay，median（IQR），d | 12（5-26） | 16(8-38) | 0.005 |
| Mortality at 28 d，n (%) | 11（32.3%） | 18(66.7%) | 0.010 |
| Mortality at 60 d，n (%) | 15（44.1%） | 18（66.7%） | 0.084 |

Abbreviations: IQR, interquartile range.

^a^The Acute Physiology and Chronic Health Evaluation (APACHE) II score ranges from 0 to 71, with higher scores indicating greater risk of hospital death. A score of 25 indicates a mortality probability of approximately 50%.

^b^The Sequential Organ Failure Assessment (SOFA) score ranges from 0 to 24, with higher scores indicating greater severity of organ dysfunction. SOFA scores between 7 and 9 are associated with a 40% to 50% mortality risk.
