# Supplementary material for: α7nAChR alleviates cognitive impairment by promoting autophagy in astrocytes via CaMKK2/AMPK/mTOR pathway in sepsis-associated encephalopathy
Source: Front Immunol. 2026 Sep 4;17:1890443. doi: 10.3389/fimmu.2026.1890443 (PMC13585608; doi:10.3389/fimmu.2026.1890443)
Supplement: Supplementary file 3 [file Table2.docx]

Table S1 Participant Characteristics

| Characteristics | Sepsis group (n = 34) | SAE group (n = 27) | P |
| --- | --- | --- | --- |
| Sex, (male/female) | 20/14 | 17/10 | 0.747 |
| Age, median (IQR), y | 70 (21-90) | 76（40-90） | 0.144 |
| Temperature, ℃ | 36.57±0.33 | 36.84±0.70 | 0.058 |
| Heart rate/min | 85.59±14.19 | 90.11±18.78 | 0.288 |
| Shock (yes/no) | 18/16 | 11/16 | 0.287 |
| APACHE II score^a^ | 16.74±6.24 | 21.42±6.46 | *0.006* |
| SOFA score^b^ | 7.30±4.39 | 8.36±4.23 | 0.343 |
| Length of ventilator，d | 5.68±4.60 | 76.2±182.5 | 0.049 |
| Length of ICU stay，median（IQR），d | 12（5-26） | 16(8-38) | 0.005 |
| Mortality at 28 d，n (%) | 11（32.3%） | 18(66.7%) | 0.010 |
| Mortality at 60 d，n (%) | 15（44.1%） | 18（66.7%） | 0.084 |

Abbreviations: IQR, interquartile range.

^a^The Acute Physiology and Chronic Health Evaluation (APACHE) II score ranges from 0 to 71, with higher scores indicating greater risk of hospital death. A score of 25 indicates a mortality probability of approximately 50%.

^b^The Sequential Organ Failure Assessment (SOFA) score ranges from 0 to 24, with higher scores indicating greater severity of organ dysfunction. SOFA scores between 7 and 9 are associated with a 40% to 50% mortality risk.

Figure S1: Relative expression levels of α7nAChR (A), GFAP (B), LC3B (C) and P62 (D) in rat hippocampus across different groups. The band intensities from western blot were normalized to GAPDH as the internal reference. n = 3 for each group. Data are presented as mean ± SD. *p < 0.05, ** p < 0.01, *** p <0.001, **** p < 0.0001; one-way ANOVA with Tukey’s post hoc test.

Figure S2: Relative expression levels of α7nAChR (A), LC3B (B), P62 (C), p-CaMKK2 (D), p-AMPK (E) and p-mTOR (F) in U118 cells after α7nAChR overexpression lentivirus transfection. The band intensities from western blot in panel A, B and C were normalized to GAPDH as the internal reference, while band intensities in panel D, E and F were normalized to β-actin as the internal reference. n = 3 for each group. Data are presented as mean ± SD. *p < 0.05, ** p < 0.01, *** p <0.001, **** p < 0.0001; one-way ANOVA with Tukey’s post hoc test.

Figure S3: Relative expression levels of α7nAChR (A), LC3B (B), P62 (C), p-CaMKK2 (D), p-AMPK (E) and p-mTOR (F) in U118 cells after α7nAChR knockdown lentivirus transfection. The band intensities from western blot in panel A, B and C were normalized to GAPDH as the internal reference, while band intensities in panel D, E and F were normalized to β-actin as the internal reference. n = 3 for each group. Data are presented as mean ± SD. *p < 0.05, ** p < 0.01, *** p <0.001, **** p < 0.0001; one-way ANOVA with Tukey’s post hoc test.
